# Evolution and Control of Imprinted *FWA* Genes in the Genus *Arabidopsis*

**DOI:** 10.1371/journal.pgen.1000048

**Published:** 2008-04-04

**Authors:** Ryo Fujimoto, Yuki Kinoshita, Akira Kawabe, Tetsu Kinoshita, Kazuya Takashima, Magnus Nordborg, Mikhail E. Nasrallah, Kentaro K. Shimizu, Hiroshi Kudoh, Tetsuji Kakutani

**Affiliations:** 1 Department of Integrated Genetics, National Institute of Genetics, Mishima, Shizuoka, Japan; 2 Department of Population Genetics, National Institute of Genetics, Mishima, Shizuoka, Japan; 3 Department of Molecular and Computational Biology, University of Southern California Los Angeles, Los Angeles, California, United States of America; 4 Department of Plant Biology, Cornell University, Ithaca, New York, United States of America; 5 Institute of Plant Biology, University of Zurich, Zurich, Switzerland; 6 Department of Biology, Faculty of Science, Kobe University, Kobe, Hyogo, Japan; The Salk Institute for Biological Studies, United States of America

## Abstract

A central question in genomic imprinting is how a specific sequence is recognized as the target for epigenetic marking. In both mammals and plants, imprinted genes are often associated with tandem repeats and transposon-related sequences, but the role of these elements in epigenetic gene silencing remains elusive. *FWA* is an imprinted gene in *Arabidopsis thaliana* expressed specifically in the female gametophyte and endosperm. Tissue-specific and imprinted expression of *FWA* depends on DNA methylation in the *FWA* promoter, which is comprised of two direct repeats containing a sequence related to a SINE retroelement. Methylation of this element causes epigenetic silencing, but it is not known whether the methylation is targeted to the SINE-related sequence itself or the direct repeat structure is also necessary. Here we show that the repeat structure in the *FWA* promoter is highly diverse in species within the genus *Arabidopsis*. Four independent tandem repeat formation events were found in three closely related species. Another related species, *A. halleri*, did not have a tandem repeat in the *FWA* promoter. Unexpectedly, even in this species, *FWA* expression was imprinted and the *FWA* promoter was methylated. In addition, our expression analysis of *FWA* gene in vegetative tissues revealed high frequency of intra-specific variation in the expression level. In conclusion, we show that the tandem repeat structure is dispensable for the epigenetic silencing of the *FWA* gene. Rather, SINE-related sequence is sufficient for imprinting, vegetative silencing, and targeting of DNA methylation. Frequent independent tandem repeat formation events in the *FWA* promoter led us to propose that they may be a consequence, rather than cause, of the epigenetic control. The possible significance of epigenetic variation in reproductive strategies during evolution is also discussed.

## Author Summary

Genomic imprinting, mono-allelic gene expression depending on the parent-of-origin, is an epigenetic process known in mammals and flowering plants. A central question in genomic imprinting is how a specific sequence is recognized as the target for epigenetic marking. In both mammals and plants, imprinted genes are often associated with tandem repeats and transposon-related sequences, but the role of these elements in epigenetic gene silencing remains elusive. *FWA* is an imprinted gene in *Arabidopsis thaliana* expressed specifically in the female gametophyte and endosperm. The *FWA* promoter is comprised of two direct repeats containing a sequence related to a SINE retroelement. Methylation of this element causes epigenetic silencing, but it is not known whether the methylation is targeted to the SINE-related sequence itself or the direct repeat structure is necessary. Here we show that the direct repeat structure is highly diverse in species within the genus *Arabidopsis*. Unexpectedly, we found that the direct repeat structure is dispensable for the epigenetic silencing and methylation of the *FWA* promoter. Rather, the SINE-related promoter sequence is sufficient for these features. Frequent independent formation of the tandem repeats suggests that they may be a consequence of the epigenetically controlled system.

## Introduction

Parental imprinting, mono-allelic gene expression depending on its parent-of-origin, is an epigenetic process known in mammals and flowering plants. In both mammals and plants, many imprinted genes are under control of DNA methylation, as their imprinting is abolished by mutations in DNA methyltransferase genes [1]–[3]. Interestingly, imprinted genes often contain sequences originated from parasitic sequences such as transposons and viruses [4]–[7]. As DNA methylation works as a defense mechanism against parasitic sequences [8]–[13], the control of imprinted genes by DNA methylation may have evolved from defense mechanisms. Another feature of imprinted genes is their frequent association with tandem repeats [14]–[17]. Despite the strength of the association, evidence is limited concerning whether the repeat structure itself is important for imprinting [15],[17].

The *FWA* gene in the flowering plant *Arabidopsis thaliana* is one of the most extensively studied systems linking the control of DNA methylation and imprinting. The *FWA* gene was originally identified through characterization of epigenetic mutants causing a heritable late-flowering phenotype. The phenotype was due to ectopic expression of the *FWA* gene in vegetative tissue [18]–[20]. In wild type plants, the *FWA* gene is silent in vegetative tissues and expressed specifically in the endosperm in an imprinted manner [3]. *FWA* silencing depends on cytosine methylation, as it is derepressed by mutations in the maintenance methylase gene *MET1* [3],[21],[22]. In addition, the imprinting is established in the female gametophyte by a “one-way” activation, which depends on the DNA demethylase DEMETER [23],[24].

In addition to these *trans*-acting components, a *cis*-requirement for the epigenetic *FWA* silencing has also been identified. Promoter of the *FWA* gene has two pairs of tandem repeats that are heavily methylated [20]. Transcription starts from this region when the methylation is lost [20]. The nucleotide sequence of this region is similar to a SINE retrotransposon [6]. Using artificial *de novo* methylation induced by double-stranded RNA, we previously showed that the critical methylated element corresponds to the SINE-related tandem repeats [25]. However, it is still unclear whether the SINE sequence *per se* can direct DNA methylation, or whether the tandem repeat structure is necessary for control of methylation and imprinted expression.

Here we investigate the evolution and natural variation of *FWA* promoters in *A. thaliana* and five other *Arabidopsis* species. The results demonstrate that the tandem repeat structure is necessary neither for imprinted *FWA* expression, nor for vegetative *FWA* silencing. Instead, the SINE-related sequence itself appears to be the primary target of the epigenetic control, which might have subsequently induced frequent tandem repeat formation during evolution. In addition, considerable intra-specific variation was found in vegetative *FWA* expression, which might present sources for epigenetic variation in reproductive strategy.

## Results

### Structure of the *FWA* Promoter in Four *Arabidopsis* Species

We have previously shown that the target of epigenetic *FWA* silencing in *A. thaliana* is a SINE-related direct repeat [25]. In order to investigate the relationship between the structure of the *FWA* promoter and *FWA* expression, we identified *FWA* orthologues in three related *Arabidopsis* species, *A. halleri*, *A. lyrata*, and *A. arenosa*. We found that the gene structure, including coding regions as well as introns and promoter sequences, was conserved among these species and *A. thaliana* (Figure S1). Notably, SINE-like sequences were found in the *FWA* promoters of all four species, suggesting that the insertion of this sequence occurred before divergence of these species.

**Figure 1:**
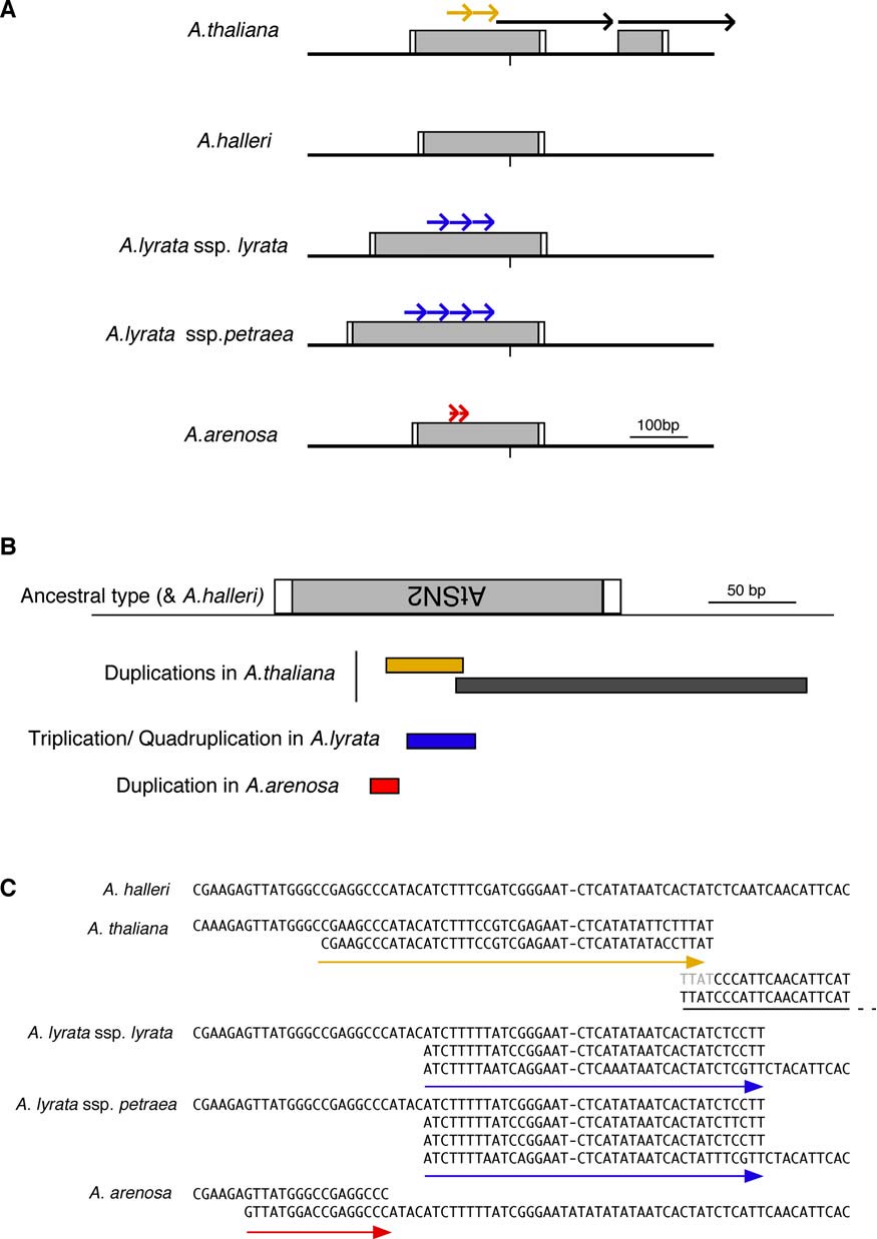
Tandem repeats in the *FWA* promoter of *A. thaliana*, *A. arenosa*, *A. lyrata*, and *A. halleri.* (A) Schematic view. Sequences with similarity to AtSINE2 are shown by gray boxes. Tandem repeats covering different regions are shown by arrows with different colors. Transcription start site is shown by vertical bar, and target site duplications of the SINE insertion are shown by white boxes. (B) Four independent repeat formation events found in the *Arabidopsis* species examined. SINE-related sequence is inserted in the orientation opposite to the transcription of *FWA*. Details of the repeats are shown in panel C. (C) Alignment of nucleotide sequences of the region in Figure 1B. Arrows indicate tandem repeats.

Unexpectedly, however, we found that duplications in the *FWA* promoters were highly diverse both within and among species (Figure 1 and Figure S2). The two pairs of tandem repeats found in *A. thaliana* were not duplicated in the other species. On the other hand, the *FWA* promoters of *A. lyrata* and *A. arenosa* contain other tandem repeats, which spanned different regions within the SINE-related sequences (Figure 1B, 1C, and Figure S2). Comparison of the structure of the *FWA* promoter of these species revealed at least four independent duplication events: two in *A. thaliana*, one in *A. arenosa*, and one in *A. lyrata*. In *A. lyrata*, the repeat number of the *FWA* promoter differs between subspecies (ssp.) *lyrata* (three copies) and ssp. *petraea* (four copies) (Figure 1 and Figure S2). In *A. halleri*, no tandem repeat was found in the *FWA* promoter (Figure 1 and Figure S2).

### Imprinted Expression

In *A. thaliana*, the *FWA* gene is silent in vegetative tissues and expressed in the endosperm in an imprinted manner (maternal-origin-specific). Both the silencing of the paternally-derived copy and silencing in vegetative tissues depend on DNA methylation in the *FWA* promoter.

**Figure 2:**
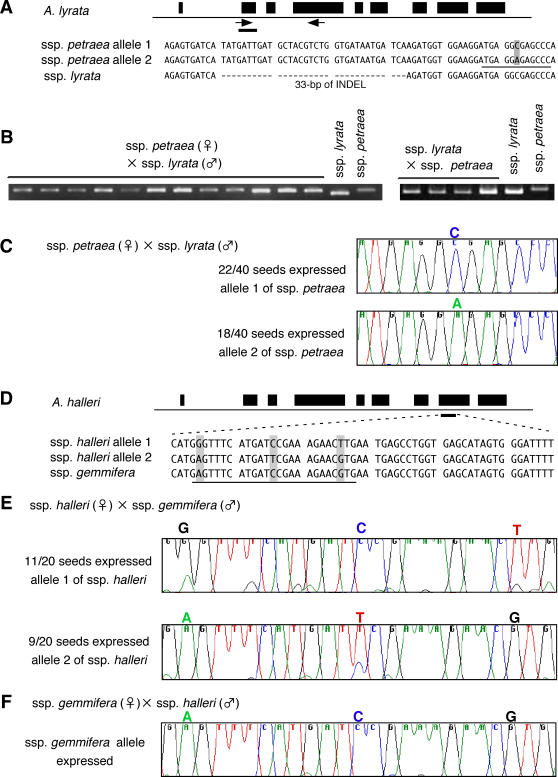
Imprinted *FWA* expression in *A. lyrata* and *A. halleri*. (A) Polymorphisms of the *FWA* gene in *A. lyrata*. Two alleles (allele 1 and allele 2) in ssp. *petraea* and one allele in ssp. *lyrata* are shown. The ssp. *lyrata* alleles have 33-bp deletion in the transcribed region compared to the *petraea* alleles. Differences between the allele 1 and 2 are indicated by a gray box. (B) RT-PCR product of *FWA* transcript in F_1_ from inter-strain crosses of both orientations. Mother and father are shown before and after x in each cross, respectively. (C) Segregation of two maternal alleles in individual immature seeds. RNA was prepared from individual immature seed and RT-PCR products were sequenced. (D) Polymorphisms of the *FWA* gene in *A. halleri*. (E) Structure of RT-PCR product from immature seeds after crosses between ssp. *halleri* (mother) and ssp. *gemmifera* (father). Plants expressing the *halleri* alleles 1 and 2 segregated. (F) Structure of RT-PCR product from immature seeds after crosses between ssp. *gemmifera* (mother) and ssp. *halleri* (father). The *gemmifera* allele was expressed.

As is the case in *A. thaliana*, *FWA* transcripts were detected in immature seeds in *A. lyrata* and *A. halleri*. To determine whether the *FWA* gene shows imprinted expression in these species, RNA was isolated from immature seeds after inter-strain crosses, and the origin of *FWA* transcripts was identified using sequence polymorphisms in the transcript. In both *A. lyrata* and *A. halleri*, the transcript detected was derived from the allele of maternal origin (Figure 2). For example, in *A. lyrata*, *FWA* transcripts of ssp. *petraea* and ssp. *lyrata* could be distinguished using polymorphisms in the transcript length (Figure 2A and 2B), and transcript of only the maternal allele could be detected in both of the reciprocal crosses (Figure 2B). The presence of maternal transcripts in seeds may reflect imprinted expression or transmission of transcripts from the female parent. In order to distinguish between these two possibilities, we used a female parent with two *FWA* alleles that could be distinguished by sequence (Figure 2A), and *FWA* transcripts were examined in 40 individual seeds. The results show segregation of the two *FWA* alleles expressed in these seeds; 22 seeds showed *FWA* RNA from one allele of the female parent and 18 seeds from the other allele (Figure 2C). This observation suggests that the *FWA* transcripts did not originate from maternal diploid tissues but from transcription of the maternally-derived alleles after meiosis, as is predicted in other imprinted genes [26]. Similarly, in *A. halleri*, we could only detect *FWA* transcripts from the maternal allele in both of the reciprocal crosses between ssp. *halleri* and ssp. *gemmifera* (Figure 2D, 2E, and 2F). In 20 individual seeds from the ssp. *halleri* x ssp. *gemmifera* cross, the 11:9 segregation of the two maternal alleles completely matches the expression pattern (Figure 2E). In summary, these results demonstrate that the *FWA* in both *A. lyrata* and *A. halleri* shows imprinted expression in immature seeds as it does in *A. thaliana*. These results are striking especially in *A. halleri*, because the *FWA* promoter of this species does not contain any tandem repeats (Figure 1). We conclude that the tandem repeat structure is not essential for the imprinting of the *FWA* gene.

### Vegetative Expression

**Figure 3:**
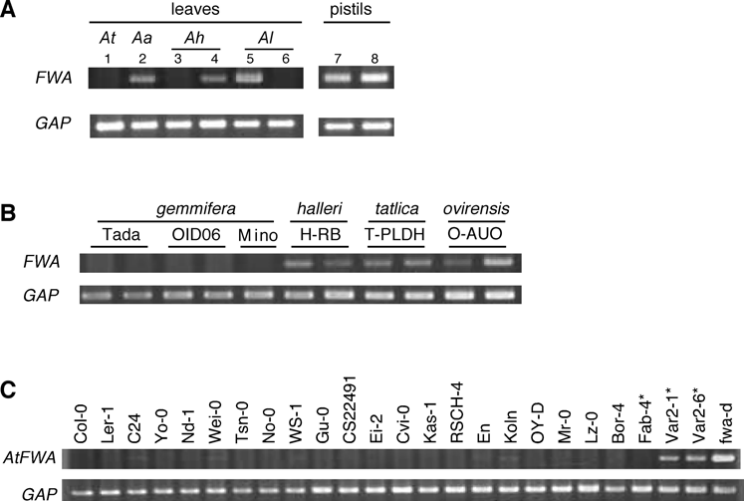
Transcription of *FWA* in leaves. (A) Vegetative *FWA* expression in *Arabidopsis* species. 1, *A. thaliana* (Col-0); 2, *A. arenosa*; 3, *A. halleri* strain Tada (ssp. *gemmifera*); 4, *A. halleri* strain RB (ssp. *halleri*); 5, *A. lyrata* pn3 (ssp. *lyrata*); 6, *A. lyrata* strain Mue-1 (ssp. *petraea*). Lanes 7 and 8 are from the same strains as lane 3 and 6, respectively. In *A. halleri* Tada (lane 3, 7) and *A. lyrata* Mue-1 (lane 6, 8), *FWA* expression was undetectable in leaves, while detectable in pistils. (B) Difference in vegetative *FWA* expression in six strains of *A. halleri*, which belong to four subspecies, ssp. *gemmifera* (Tada, OID06, Mino), ssp. *halleri* (H-RB), ssp. *tatrica* (T-PLDH), and ssp. *ovirensis* (O-AUO). Although vegetative *FWA* transcription was not detectable in most natural isolates of ssp. *gemmifera*, it was consistently detectable in other isolates (Figure S3). (C) Vegetative *FWA* expression in 24 natural strains of *A. thaliana*. An asterisk indicates natural strains without the 45-nt tandem repeat. Two to four plants were examined for each strain and gave consistent results. *fwa-d* is a late flowering epigenetic allele induced in a *ddm1* mutant background [19].

We next examined vegetative *FWA* expression in these species. Since *A. halleri* does not have tandem repeats in its *FWA* promoter, we were able to test whether the tandem repeat is necessary for vegetative silencing. In contrast to *A. thaliana*, in which the *FWA* gene is silent in vegetative tissues [20], a low level of *FWA* transcript was often detected in the vegetative tissues of *A. arenosa*, *A. lyrata*, and *A. halleri* (Figure 3). Interestingly, vegetative *FWA* expression shows considerable variation at least at the subspecies level. We could detect the vegetative *FWA* transcript in two strains of *A. lyrata* ssp. *lyrata* but not in a strain of *A. lyrata* ssp. *petraea* (Figure 3A, lanes 5 and 6). Similarly, we could detect vegetative *FWA* transcripts in *A. halleri* ssp. *halleri*, *tatrica*, *ovirensis*, but not in nine out of eleven strains of ssp. *gemmifera* (Figure 3B and Figure S3). Therefore, the tandem repeat structure is not essential for epigenetic silencing of *FWA* in vegetative tissues.

The vegetative *FWA* silencing observed in strains of *A. halleri* ssp. *gemmifera* and *A. lyrata* ssp. *petraea* does not seems to be due to loss of promoter function, because their *FWA* gene was expressed in immature seeds (Figure 2F) and in pistils (Figure 3A right panel), which contain female gametophytes. Thus, the vegetative *FWA* silencing is likely to have an epigenetic basis. The variation in the vegetative *FWA* expression was heritable; the *FWA* gene remained silent in the self-pollinated progeny of those plants with silent *FWA*, while vegetative *FWA* expression was detected in other strains growing in parallel (not shown).

### Allotetraploids and Inter-Specific Hybrids

We further examined *FWA* expression in two allotetraploid species, *A. kamchatica* (ssp. *kamchatica* and *kawasakiana*) and *A. suecica*. The former is an allotetraploid between *A. lyrata* and *A. halleri* [27], and the latter between *A. arenosa* and *A. thaliana* [28].

**Figure 4:**
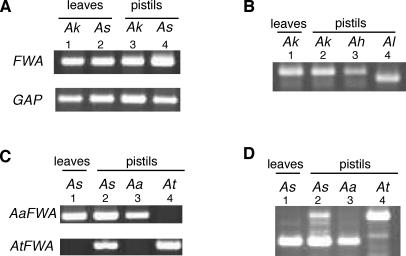
*FWA* transcripts in allotetraploids. (A) Vegetative *FWA* transcript in *A. kamchatica* ssp. *kamchatica* strain FJSB1 (*Ak*) and *A. suecica* JS7 (*As*). (B) In the *A. kamchatica*, only the *halleri*-type copy was transcribed in both leaves and pistils (transcript of the *lyrata*-type copy was undetectable). The *halleri*-type and *lyrata*-type copies were distinguished by length after *Rsa*I digestion of the PCR products. *Ah* and *Al* in lanes 3 and 4 are samples isolated from *A. halleri* Tada and *A. lyrata* pn3, respectively. (C and D) In *A. suecica*, only the *arenosa*-type copy was transcribed in leaves, but both *arenosa*- and *thaliana*-type copies were transcribed in pistils, suggesting that the *thaliana*-type copy was epigenetically silent in leaves. These two types of transcripts were distinguished using specific primer pairs (C) or by restriction digestion (D). See Materials and methods for details.

We were able to detect vegetative *FWA* expression in *A. kamchatica* (ssp. *kamchatica* and *kawasakiana*) (Figure 4A lane 1). As expected from its allotetraploid origin, *A. kamchatica* has two copies of *FWA* genes, with one copy being structurally similar to the *A. lyrata FWA* gene and the other similar to the *A. halleri FWA* gene (Supplementary Figure S4A and S4C). We examined the expression of each copy using polymorphisms between them. In both ssp. *kamchatica* and ssp. *kawasakiana*, the *A. halleri*-like *FWA* copy was expressed in leaves, while the *A. lyrata*-like copy was silent (Figure 4B lane 1). Interestingly, the *lyrata*-type copy was also silent in pistils, which contain the female gametophytes (Figure 4B lane 2), leaving open the possibility that the silencing of the *lyrata*-type copy was not epigenetic but genetic (for example, by a mutation in the promoter).

We were also able to detect vegetative *FWA* expression in *A. suecica* (Figure 4C lane 1), an allotetraploid between *A. arenosa* and *A. thaliana*. As expected, *A. suecica* has two copies of *FWA* genes, which are structurally similar to the *FWA* gene of either *A. arenosa* or *A. thaliana* (Figure S4B and S4C). Vegetative *FWA* expression of *A. suecica* was mainly from the *arenosa*-type copy; we could not detect vegetative expression of the *thaliana*-type copy (Figure 4C and 4D). On the other hand, both *arenosa*-type and *thaliana*-type copies were expressed in pistils (lane 2 of Figure 4C and 4D). Therefore, the *thaliana*-type copy was specifically silenced in vegetative tissues, as is the case in the parental species *A. thaliana*.

**Figure 5:**
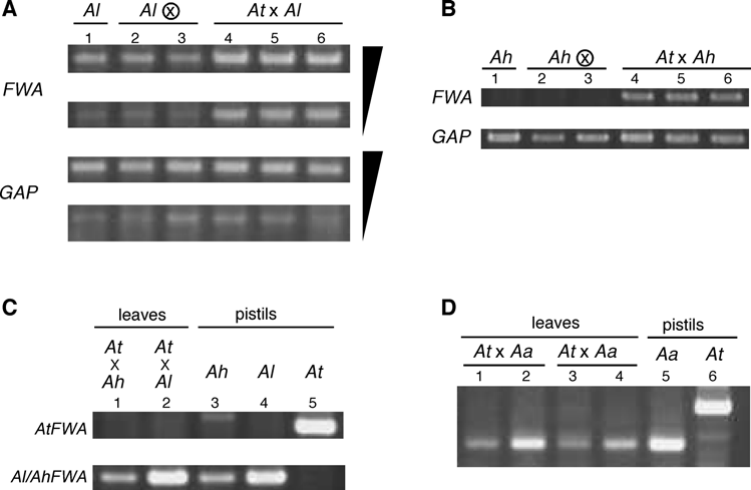
*FWA* transcripts in synthetic hybrids. (A) F_1_ progeny from a cross between *A. lyrata* pn3 and *A. thaliana ms-1* mutant. The F_1_ hybrids (lanes 4–6) showed increased *FWA* transcripts compared to their direct parent (lane 1) and its self-pollinated progeny (lanes 2, 3). For both *FWA* and *GAP*, lower panels are from PCR reactions with three cycles less than those in the upper panels. The *ms-1* mutant was used as the female parent for efficient crossing. Essentially the same results were obtained when wild type Col plant was used for the female parent, or when *A. lyrata* MN47 was used as the male parent. (B) F_1_ progeny from a cross between *A. halleri* Tada (ssp. *gemmifera*) and *A. thaliana ms-1* mutant. The F_1_ plants showed *FWA* expression, while this gene is silent in their parents. (C) The *FWA* expressed in the F_1_ hybrid was from *lyrata*–type or from *halleri*-type. The *FWA* genes of the *thaliana*-type and from *lyrata*– or *halleri*-type were distinguished using specific primer pairs for the RT-PCR (see Materials and Methods for details). The *FWA* gene of *thaliana*-type remained silent in the leaves of the hybrids (lane 1, 2). (*D*) Expression of *FWA* in synthetic hybrids between *A. arenosa* and *A. thaliana* (L*er* for the lane 1, 2 and Col for the lane 3, 4). The *FWA* gene of *thaliana*-type remained silent in the leaves of the hybrids.

In the allotetraploids, vegetative *FWA* expression tends to be stronger than that in their parental species. In order to evaluate the direct effects of hybridization, we examined *FWA* expression in artificially generated inter-specific hybrids. In hybrids between *A. thaliana* and *A. lyrata* ssp. *lyrata,* expression level of the *lyrata FWA* in leaves was increased significantly compared to that in its direct parent *A. lyrata* ssp. *lyrata* (Figure 5A and 5C). In hybrids between *A. thaliana* and *A. halleri* ssp. *gemmifera* strain Tada, the *halleri*–like copy was expressed (Figure 5B and 5C). This result is striking considering that the *FWA* was completely silent in vegetative tissues of the parent strain Tada (Figure 5B, lane 1–3). On the other hand, in both the *A. lyrata-A. thaliana* and *A. halleri-A. thaliana* hybrids, the *A. thaliana* copy remained silent (Figure 5C). In addition, in the synthetic allotetraploid of *A. arenosa* and *A. thaliana* (synthetic *A. suecica*; reference [29]), the *arenosa*-type copy was expressed while the *thaliana*-type copy was silent (Figure 5D). In summary, the *FWA* genes tend to be transcriptionally activated in the inter-specific hybrids. This phenomenon might be related to previous finding that the imprinted genes, such as *PHERES1* and *MEDEA,* show abnormal expression patterns in hybrids between *A. thaliana* and *A. arenosa* [30]. In contrast to the expression of the *lyrata-*, *halleri*- or *arenosa*-derived *FWA* copies in the hybrids, *thaliana*-derived copies remained silent in all the hybrids. These observations are consistent with that in the situation in natural allotetraploids; the *thaliana*-derived copy was most stably silenced in the vegetative tissues of both the allotetraploids and hybrids.

### DNA Methylation

**Figure 6:**
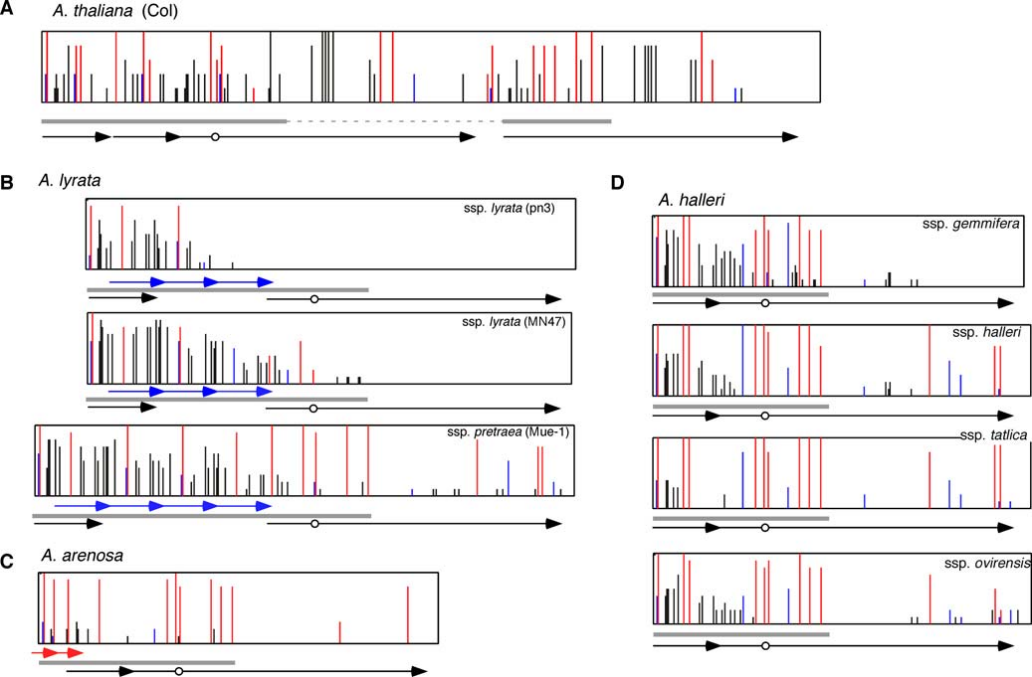
Cytosine methylation status of the *FWA* promoter. Ten clones from bisulfite-treated templates were examined for each sample. Red, blue, and black bars represent methylation in CG, CNG, and asymmetric sites, respectively. Blue and red-arrows shows *lyrata*-specific and *arenosa*-specific tandem repeats, respectively. Black arrows show regions duplicated in *A. thaliana*. Gray bars show the SINE-related sequences. The circle shows the transcription start site. *A. thaliana* used was strain Col. *A. halleri* ssp. *gemmifera* used was strain Tada. Strains of the other subspecies of *A. halleri* were those used in Figure 4.

In *A. thaliana*, loss of DNA methylation in the *FWA* promoter induces release of epigenetic silencing. The tandem repeat is methylated in all 96 natural *A. thaliana* strains [31]. The methylated region of the *FWA* promoter precisely matched the tandem repeat regions and the methylation extends out of the SINE-related region to the end of the repeat [20] (Figure 6A). We were therefore interested in whether the epigenetic silencing of the *FWA* is also related to DNA methylation in *Arabidopsis* species with less prominent repeat structure.

Figure 6 and Table S1 show bisulfite-mediated genomic sequencing data demonstrating that the SINE-related region of the *FWA* promoter was methylated in all species examined, including *A. halleri*, indicating that the repeat structure is not necessary for directing DNA methylation to this region. CG-sites tend to be more heavily methylated than non-CG sites, as is the case in the *FWA* promoter of *A. thaliana*.

The methylation level is high throughout the repeat region *in A. thaliana*, which shows the most stable silencing (Figure 6, Table S1). Interestingly, intraspecific variation in the methylation level was found in *A. lyrata* (Figure 6B). The methylation level tended to be negatively correlated with the expression level; it was highest in the strain Mue-1 (ssp. *petraea*), which shows vegetative silencing of the *FWA*.

### Variation of the *FWA* Promoter within *A. thaliana*

**Figure 7:**
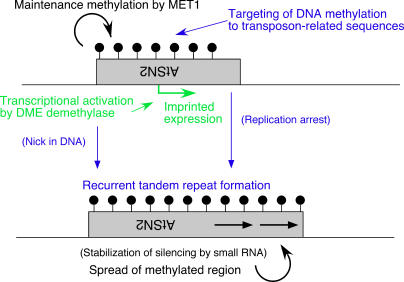
A speculative model explaining the origin and role of tandem repeats and SINE-related sequences in the imprinted gene *FWA*. Developmental processes are shown in green, and proposed evolutionary events in blue. See text for details.

In *A. lyrata*, the number of repeats in the *FWA* promoter correlated with the level of vegetative silencing. In *A. lyrata* ssp. *petraea* strain Mue-1, which has four copies of the tandem repeat, the vegetative expression was much lower than that in the two strains of *A. lyrata* ssp. *lyrata* that have three copies of the tandem repeat (Figure 3A and Figure S4A). The silencing also correlated with high level of promoter methylation (Figure 7B).

In order to test whether the correlation between copy number in the repeats and vegetative *FWA* silencing is found also in *A. thaliana*, we examined the structure of the *FWA* promoter and its expression in 96 natural strains of this species. All 96 strains had the long repeat, but the short repeat was not found in three strains, Fab-4, Var2-1, and Var2-6. We then examined vegetative *FWA* expression in these strains and, as controls, 21 strains with two pairs of tandem repeats (Figure 3C). Vegetative *FWA* expression was not detected in any of the 21 control strains, while two out of three strains lacking the short repeat showed a low level of vegetative *FWA* expression.

## Discussion

### Tandem Repeat Structure and the SINE-Related Element

Here we reported variation in the structure and expression of the imprinted gene *FWA* in the genus *Arabidopsis*. The promoter sequence related to a SINE retroelement was found in the *FWA* locus of all *Arabidopsis* species examined. Most unexpectedly, the tandem repeat structure in this region is not essential for the epigenetic silencing of the *FWA* gene. The *FWA* promoter of *A. halleri* does not have the repeat structure, but it shows imprinted expression and vegetative silencing.

Similarly, tandem repeats are often found in CpG islands of mammalian imprinted genes, but they are not always conserved between mouse and human [32]. In addition, deletion of a conserved direct repeat element upstream of H19 had no effect on imprinting [15]. One possible interpretation of these observations is that tandem duplications may be a consequence, rather than the cause, of mono-allelic expression. Consistent with this interpretation, we found four independent duplication events in the small region of the *FWA* promoter in closely related species, *A. thaliana*, *A. lyrata*, and *A. arenosa*.

Silencing of the *FWA* gene tends to be stronger in *A. thaliana* than in other species. The majority of *A. lyrata* and *A. halleri* strains showed vegetative *FWA* expression. Vegetative *FWA* expression was also found in examined strains of *A. arenosa*, *A. kamchatica* and *A. suecica*. On the other hand, the *FWA* gene in *A. thaliana* was silent in all the examined 21 accessions that have two direct repeats. Vegetative *FWA* expression tends to be elevated after inter-species hybridization, the clearest example being the *FWA* gene of the *A. halleri* strain Tada. Although we could not detect vegetative transcripts in this strain, *FWA* transcript was detected after hybridization with *A. thaliana*. On the other hand, vegetative expression was not detectable for the *A. thaliana*-derived *FWA* gene, even after hybridization with *A. halleri*, *A. lyrata*, or *A. arenosa*. The *thaliana-*type *FWA* was also silent in the natural allotetraploid *A. suecica*.

Stable silencing of the *A. thaliana FWA* in vegetative tissues might depend on the presence of tandem repeats in the promoter. Consistent with this conjecture, vegetative *FWA* expression, although at very low levels, was found in two of three natural accessions that do not have one of the two tandem repeats (Figure 3). The tandem direct repeats may facilitate the production of small RNA and targeting of DNA methylation to stabilize silencing [33]. Small RNA was detected in the *FWA* promoter of *A. thaliana* [6]. In this context, it might be interesting to see if small RNA is detectable corresponding to the *FWA* promoter of *A. halleri*, which does not have the tandem repeat. Results of modified transgene with various repeat structures of the *FWA* promoter suggest that the tandem repeat structure is effective in inducing de novo DNA methylation at least in a transgenic system [34].

Based on these observations, we propose a model for the evolution of the epigenetically controlled *FWA* gene (Figure 7). Tandem repeat structure does not appear to be essential for the imprinted expression, vegetative silencing, or targeting of DNA methylation in the *FWA*. Rather, methylation is directed to the SINE-related sequence, which functions as the core of the local heterochromatin. Subsequently, tandem repeats have been recurrently generated in this region during evolution. Repeat formation might be caused after replication arrest [35] in the epigenetically silent region. In addition, DEMETER DNA demethylase might induce tandem repeat by nick formation [23] when expressed in the embryonic cell lineage [36]. DNA methylation would have then spread to the region of the tandem repeat, which stabilizes epigenetic silencing. The methylation, especially in CG sites, function as an epigenetic mark heritable over multiple generations (Figure 7).

### *FWA* as a Possible Epigenetic Flowering-Time Modifier

Heritable epigenetic variation is an enigmatic genetic phenomenon, which is known both in mammals and plants [37],[38]. The *FWA* gene in *Arabidopsis* has the potential to cause epigenetic variation in flowering time, and natural variation in vegetative *FWA* expression was found in this study (Figure 3).

Sequence analysis of the *FWA* promoter in 96 natural strains of *A. thaliana* revealed a high level of variation in this region (Table S2 and Figure S6). C/G to T/A mutations are overrepresented, possibly reflecting high mutation rate in the methylated C to T [39],[40]. Despite the high mutation rate, alleles of intermediate frequency are significantly underrepresented in this type of variation (Tajima's D: −1.7078, P<0.05), suggesting that new mutations were rapidly eliminated (Table S2). Negative selection against mutation in the C/G sites may reflect an advantage of stable silencing of the *FWA* gene by cytosine methylation, especially in *A. thaliana*, which has a rapid life cycle.

Unlike *A. thaliana*, other *Arabidopsis* species showed vegetative *FWA* expression in the majority of strains. The expression level was heritable but variable within species (Figure 3 and Figure S3). The rapid and potentially reversible changes in epigenetic states might represent an important source of variation in reproductive strategy.

## Materials and Methods

### Plant Materials

The sources of the 96 natural accessions (cs22660) of *A. thaliana* are described in Nordborg et al. [41]. The epigenetic late-flowering *fwa* mutant was induced in the *ddm1-1* background and backcrossed to *DDM1/DDM1* background as described previously [25]. The male-sterile mutant (*ms1-1*) is a kind gift from Maarten Koornneef. *A. lyrata* ssp. *lyrata* pn3 was collected in Pores Knob, Wilkes County, NC. The *A. lyrata* ssp. *lyrata* MN47 was developed at Cornell University from material originally isolated by Charles Langley in Michigan, USA. The *A. lyrata* ssp. *petraea* strain Mue-1, was collected in Muehlberg, Germany. *A. halleri* ssp. *gemmifera* Tada, OID06-6, and Mino were isolated in Inagawa-cho, Osaka, Japan, Taka-cho, Hyogo, Japan, and Mino-shi, Osaka, Japan. *A. halleri* ssp. *halleri* H-RB was isolated in Bistrita, Romania, ssp. *tatrica* T-PLDH1 in Vysoke Tatry, Poland, ssp. *ovirensis* O-AUO26 in Karinthia, Austria. *A. kamchatica* ssp. *kamchatica* FJSB1 was isolated in Subashiri, Shizuoka, Japan, and ssp. *kawasakiana* Shirahama was isolated in Ohmi-shirahama, Shiga, Japan. *A. suecica* was from Sendai Arabidopsis Seed Stock Center (JS7). Synthetic *suecica* (CS22665 and CS22666) and *A. arenosa* (CS3901) are from Arabidopsis Biological Resource Center at Ohio State University.

### Cloning and Sequencing of DNA Fragments of *FWA*

Sequences of all primers used are shown in Supplementary Text S1. Genomic DNAs were isolated from leaves by Nucleon (Amersham Biosciences, UK). The DNA fragments spanning the *FWA* gene in related species were amplified by PCR and cloned into pGEM-TEasy or pCR2.1-TOPO vector using the primers lyrata-7f and lyrata-10r for lyrata and arenosa alleles, lyrata-7f+lyrata-9r for halleri alleles, FWApro1+As-3 for thaliana alleles. In each strain, multiple clones were sequenced and regions of inconsistent sequences were directly sequenced from the genomic DNA again. Sequences of both alleles were determined in out-crossing species, such as *A. halleri* and *A. lyrata*. 96 ecotypes of the tandem repeat region was amplified using primer pair Ateco96F+Ateco96R, and their nucleotide sequences were determined directly from the genomic DNA. Data were analyzed using Sequencher (Gene Codes Corporation, MI, USA). Harr plot analysis was performed with GENETYX-MAC software (Software Development Co., LTD, Tokyo, Japan). Unit size to compare was 10 bp, and dot plot matching number was 9 bp [42]. The sequence alignment was made using ClustalW (http://www.ddbj.nig.ac.jp/search/clustalw-j.html). A phylogenetic tree was constructed by the neighbor joining method with K2P distance [43], and bootstrap probabilities of 1,000 trials were calculated.

### Gene Expression Analysis

Total RNA was isolated from leaves and pistils from open flowers using SV Total RNA Isolation system (Promega, WI, USA). From 2 µg total RNAs, first strand cDNA was synthesized using random primers by a First-strand cDNA synthesis Kit (Amersham, NJ, USA). *FWA* transcript was detected by RT-PCR of 40 cycles using the first strand cDNA as a template with primer pair FWA-RT-F2+FWA-RT-R1. GAPC was used as control and amplified by RT-PCR of 25 cycles using primer pair GAP3+GAP5. In *A. suecica*, allele-specific RT-PCR was performed using primer pair specific for *FWA* in *A. thaliana* (AtFWA-RT-F1+AtFWA-RT-R1) and specific for *FWA* in *A. arenosa* (FWA-RT-F5+AtFWA-RT-R4). In hybrid between *A. lyrata*/*A. halleri* and *A. thaliana*, allele specific primer pair was used for RT-PCR to amplify *FWA* in *A. lyrata*/*A. halleri* (FWA-RT-F5+AtFWA-RT-R5) and that of *A. thaliana* (AtFWA-RT-F1+AtFWA-RT-R1). The PCR condition was 95°C for 10 sec followed by 25 or 40 cycles of 95°C for 30 sec, 57°C for 30 sec, and 72°C for 30 sec.

Total RNAs from one premature fertilized seed after 15DAP in *A. lyrata* and *A. halleri* were isolated by RNAqueous-Micro (Ambion, Texas, USA). Using all of isolated total RNAs, first strand cDNA was synthesized using *FWA-*specific primer AlFWAcDNA-R by SuperScript III Reverse Transcriptase (Invitrogen). *FWA* transcripts in seed of *A. lyrata* and *A. halleri* were amplified by RT-PCR of 35 cycles using the first strand cDNA as a template with primer pairs (AhAlFWAcDNA-F2+lyrata2r) and (FWA-RT-F3+FWA-RT-R3), respectively. Products from genomic DNA and mRNA could be distinguished by size.

*FWA* transcript level was compared between two strains of *A. kamchatica* by real-time PCR (Figure S5) with the *GAP* gene used as the internal control. The cDNA was amplified using SYBR Premix Ex Taq (Takara Biomedicals, Japan) on a LightCycler (Roche, Rotkreuz, Switzerland). With the primer pairs FWAreal1+FWA-RT-R1 (*FWA*) and GAPreal2+GAPreal3 (*GAP*). The PCR condition was 95 for 10sec followed by 25 (*GAP*) or 40 (*FWA*) cycles of 95°C for 5 sec, 55°C for 10 sec, and 72°C for 7 sec. Data were analyzed with LightCycler Software version 3.5 (Roche).

### Detection of DNA Methylation by the Bisulfite Method

Bisulfite sequencing was performed according to Paulin et al. [44]. After chemical bisulfite reaction, PCR fragments of repeat region of *FWA* were amplified using primer pairs as follow. AtFWA; (AtFWA-Bis-F1+AtFWA-Bis-R1), AaFWA; (AhFWA-Bis-F1+arenosaRTbisR), AlFWA; (arenosaRTbisF+lyrataRTbisR), and AhFWA; (AhFWA-Bis-F1+AhFWA-Bis-R2). The amplified PCR fragments were gel purified by GENECLEAN III Kit (Q-Biogene, CA, USA) and cloned into pGEM-T easy vector (Promega, WI, USA), and 10 independent clones were sequenced.

### Accession Numbers

Sequence data from this article have been deposited with DDBJ/GenBank/EMBL libraries under accession numbers AB363659–AB363674, AB367805–AB367817, and AB367818–AB367913.

## Supporting Information

Text S1 Supplementary materials and methods.
(0.03 MB PDF)

Figure S1 FWA genes in Arabidopsis species. Harr plot analysis shows that not only exons (black boxes) but also promoter and introns are conserved. A big gap found in each panel reflects thaliana-specific big tandem duplication. Gray boxes indicate the regions related to the SINE.
(0.05 MB PDF)

Figure S2 Harr plot analysis of 5′ region of the *FWA* gene to detect tandem repeats. Black boxes and gray bar indicate exons and the SINE-related region, respectively. In the SINE-related regions, two, three, and four tandem repeats were found in *A. arenosa*, *A. lyrata* ssp. *lyrata*, and *A. lyrata* ssp. *petraea*, respectively. Two pairs of tandem repeats were found in *A. thaliana*. No tandem repeat was found *A. halleri*.
(0.08 MB PDF)

Figure S3 Variation in vegetative *FWA* expression in eleven plants of *A. halleri ssp. gemmifera* isolated around Kyoto area in Japan. (A) The vegetative *FWA* expression was detectable in two isolates, while it was undetectable in the other nine isolates. (B) Same expression pattern was detected in different individuals isolated from the same area. (C) Map of the locations these plants were isolated. Abbreviation of the isolates: AK (Akebe), MH (Mikohata), IK (Ikuno), OM (Omoidegawa, Taka), RK (Rokko), TD (Tada), MK (Myouken), MN (Mino), OH (Ohara), IB (Mt. Ibuki), OE (Mt. Ooe). The plants were isolated from the field and were grown in the laboratory. Three of them, Mino, Omoidegawa, and Tada, were further characterized after isolation of the seeds and growth in the laboratory (Figure 3B).
(0.12 MB PDF)

Figure S4 Structure of *FWA* genes in allotetraploids and parental species. (A) Two *FWA* copies in *A. kamchatica*. Each of them was similar to that in *A. halleri* (left panel) or *A. lyrata* (right panel). The similarity was found not only in exons (black boxes) but also promoter and introns. Gray boxes indicate the SINE-related regions. Copy number of tandem repeats in the lyrata-like copy was three as is the case in ssp. *lyrata*. (B) Two *FWA* copies in *A. suecica*. Each of them was similar to that in *A. thaliana* (left panel) or *A. arenosa* (right panel). (C) A phylogenetic tree of nucleotide sequences of full length cDNA of *FWA* in the *Arabidopsis* species constructed by the neighbor-joining method. *Arabis glabra FWA* was used as the outgroup. Bootstrap values with 1,000 repeats are indicated at the nodes of the neighbor-joining tree.
(0.08 MB PDF)

Figure S5 Differences in promoter structure and expression pattern of the halleri-type *FWA* gene in two strains of in *A. kamchatica*, SHR1 (ssp. *kawasakiana*), and FJSB1 (ssp. *kamchatica*). (A) Promoter alignment revealed insertion of a possible transposable element in the SHR1 allele. Blue and red letters indicate the predicted target site duplication and terminal inverted repeat, respectively. This insertion was found in majority of *A. kamchatica*, ssp. *kawasakiana* strains isolated in Japan (not shown). Gray boxes: the SINE-related sequences. Green letter shows the target site duplication of the SINE insertion. The circle shows the transcription start site. Black arrows show regions duplicated in *A. thaliana*. (B) Methylation status of the halleri-type *FWA* gene in the two strains. The predicted transposon (white box) was heavily methylated in SHR1. (C) Expression analysis of the *FWA* gene. Related *FWA* expression level measured by real-time PCR are shown below the gel. The transposon insertion and increase in the DNA methylation correlates with reduction in the *FWA* transcript level in SHR1.
(0.04 MB PDF)

Figure S6 Sliding window analyses of silent divergence and diversity in the *FWA* gene. The analyses are based on the 12 strains of *A. thaliana*. K and p in 200 bp silent sites are plotted in 1 bp intervals. Under the sliding window plot, exons (from third to last) are shown by black boxes and repeated regions by arrows.
(0.02 MB PDF)

Table S1 Proportion of methylated cytosine around the *FWA* promoter.
(0.04 MB PDF)

Table S2 Patterns of nucleotide variation in the *FWA* gene within *A. thaliana*.
(0.05 MB PDF)
