# Supplementary material for: Evolution and Control of Imprinted FWA Genes in the Genus Arabidopsis
Source: PLoS Genet. 2008 Apr 4;4(4):e1000048. doi: 10.1371/journal.pgen.1000048 (PMC2270340; doi:10.1371/journal.pgen.1000048)
Supplement: Text S1 — Supplementary materials and methods. (0.03 MB PDF) [file pgen.1000048.s001.pdf]

Supplementary Materials and Methods.

Sequences of the primers used. The sequences are shown in the order appeared in the Materials methods in the main text.

(lyrata-7f+lyrata-10r)
5’-TCATATGAACTCATAAAGAAGAGT-3'
+5’-TGGCACATGCCAATGTCGA-3'

(lyrata-7f+lyrata-9r)
5’-TCATATGAACTCATAAAGAAGAGT-3'
+5’-ATCCGGAGGAGTCTTGA-3'

(FWApro1+As-3)
5’-RGAATCAAKTGKRTTTAGTGTTTAC-3'
+5’-CAACGGAAACTGGAGCTGCTGATGG-3'

(Ateco96F+Ateco96R)
5’-AACTAGGCCATCCATGGATGGTTTC-3'
+5’-AATTCAAGTAGAGAATGAACCAG-3',

(FWA-RT-F2+FWA-RT-R1)
5’-AAAAGTGGCTCACTCCAACWGATTC-3'
+5’-CAMGTTCTTTCGGATCATGAAACTC-3'.

(GAP3+GAP5)
5’-CACTTGAAGGGTGGTGCCAAG-3'
+5’-CCTGTTGTCGCCAACGAAGTC-3'.

(AtFWA-RT-F1+AtFWA-RT-R1)
5’-AGCTGAAGGCGATGAGATTGATATG-3'
+5’-CTTTGTTTGGGATGAGAAAGGTATC-3'

(FWA-RT-F5+AtFWA-RT-R4)
5’-CAAATCCAAGCGGAGCTGAAAATGG-3'
+5’-TAGGCTCCATATTAAGCCTTTGTCC-3'.

(FWA-RT-F5+AtFWA-RT-R5)
5’-CAAATCCAAGCGGAGCTGAAAATGG-3'
+5’-GGGACGGGAAGGGATTTTGGAATAC-3'

(AtFWA-RT-F1+AtFWA-RT-R1)
5’-AGCTGAAGGCGATGAGATTGATATG-3'
+5’-CTTTGTTTGGGATGAGAAAGGTATC-3'.

primer (AlFWAcDNA-R)
5’-TTATGTATATATATGTAACGCGG-3'

(AhAlFWAcDNA-F2+lyrata2r)
5’-ATGAATGAACAAGGTGATGATTTTG-3'
+5’-CTGGTGTTGCATTTATTCCTGGA-3'

(FWA-RT-F3+FWA-RT-R3)
5’-TTGAACAAGCGGAATATAATGAGAG-3'
+5’-ATGCATCATTCCAAATCTCTTGCAG-3',

(FWAreal1+FWA-RT-R1)
5’-ACARCGAATGACTCTCAACTACTAC-3'
+5’-CAMGTTCTTTCGGATCATGAAACTC-3'

(GAPreal2+GAPreal3)
5’-ATCAACAGTCTTCTGAGTAGCAGTG-3'
+5’-TTGCTCCCCTTGCCAAGGTTATCAA-3'.

(AtFWA-Bis-F1+AtFWA-Bis-R1)
5’-TATAYTAATATYAAAGAGTTATGGG-3'

+5’-CRRRAACCAAAATCATTCTCTAAACA-3',

(AhFWA-Bis-F1+arenosaRTbisR)
5’-TAYAYTTATATYGAAGAGTTATGGG-3'
+5’-TAGTTTAGAGGATGATTAYGGTTTYTGA-3',

(arenosaRTbisF+lyrataRTbisR)
5’-AGTGAGGAATYAAGTGTATTTAGTGTT-3'
+5’ACARACAATCARAAACCATTCTAAACCA-3',

(AhFWA-Bis-F1+AhFWA-Bis-R2)
5’-TAYAYTTATATYGAAGAGTTATGGG-3'
+5’-CCATAATCATCCTCTAAACCAAATAT-3'.
