# Supplementary figures and images for: Evolution and Control of Imprinted FWA Genes in the Genus Arabidopsis
Source: PLoS Genet. 2008 Apr 4;4(4):e1000048. doi: 10.1371/journal.pgen.1000048 (PMC2270340; doi:10.1371/journal.pgen.1000048)

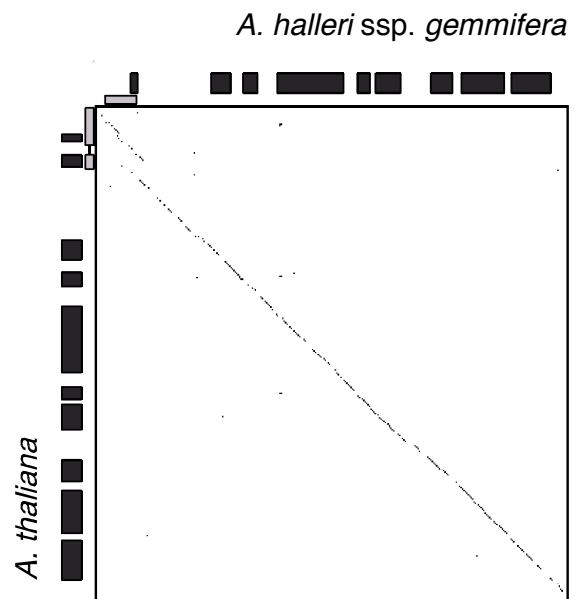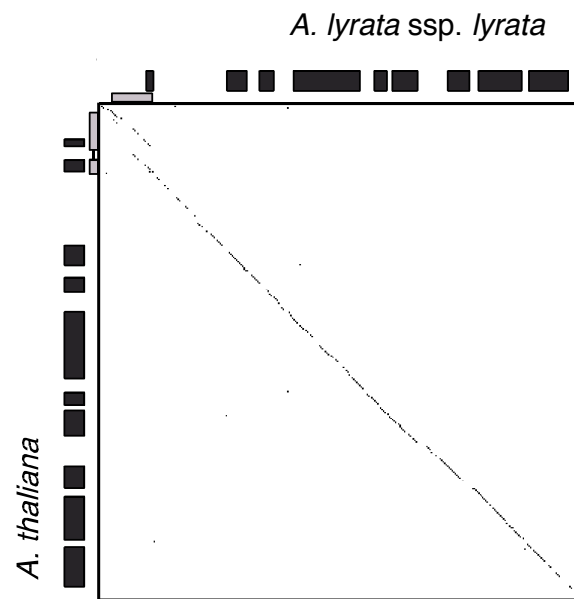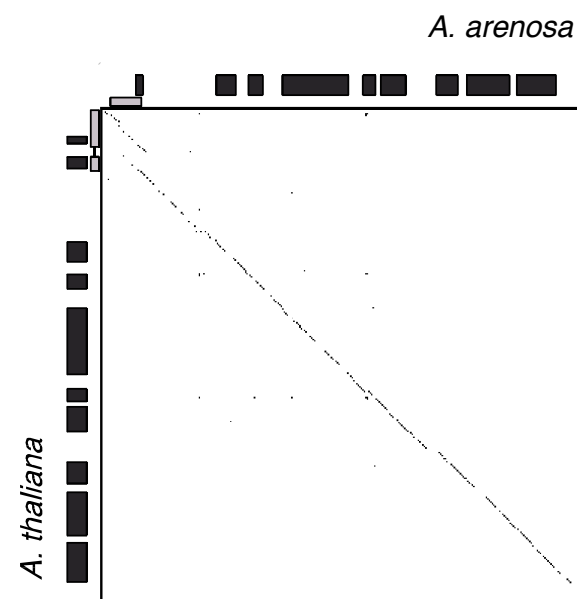

Supplement: Figure S1 — FWA genes in Arabidopsis species. Harr plot analysis shows that not only exons (black boxes) but also promoter and introns are conserved. A big gap found in each panel reflects thaliana-specific big tandem duplication. Gray boxes indicate the regions related to the SINE. (0.05 MB PDF) [file pgen.1000048.s002.pdf]

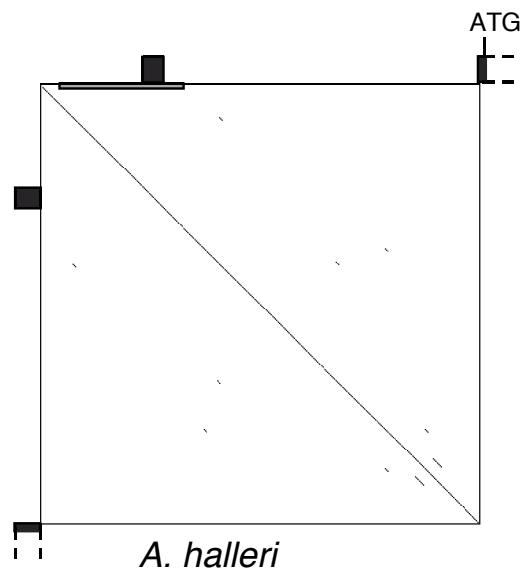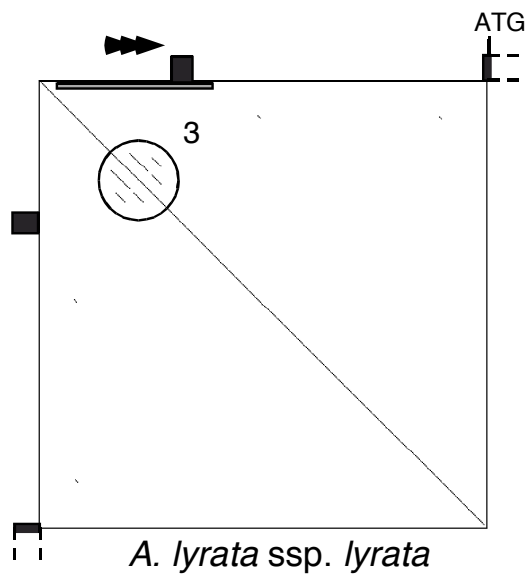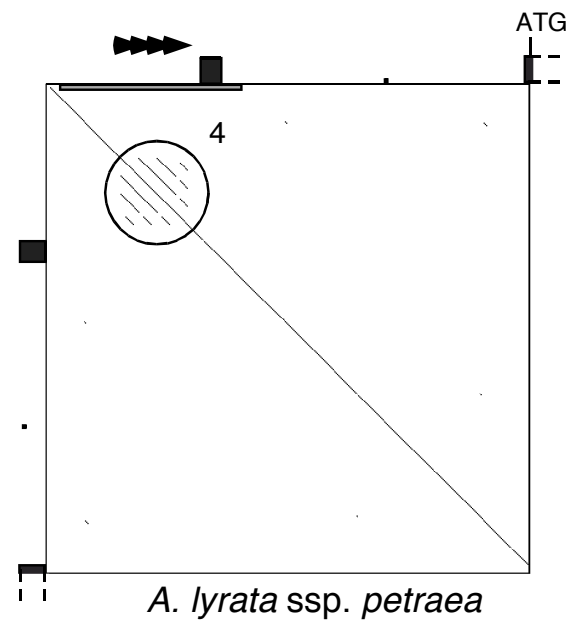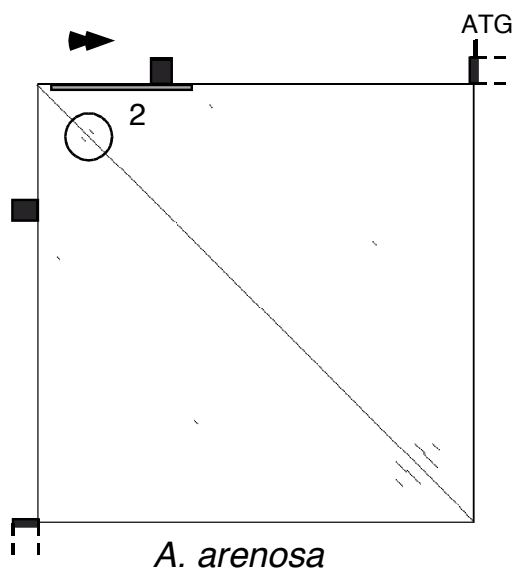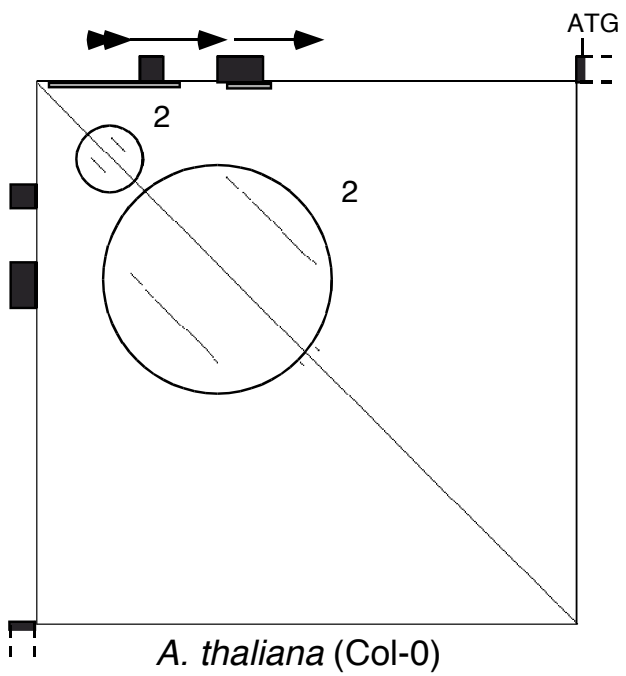

Supplement: Figure S2 — Harr plot analysis of 5′ region of the FWA gene to detect tandem repeats. Black boxes and gray bar indicate exons and the SINE-related region, respectively. In the SINE-related regions, two, three, and four tandem repeats were found in A. arenosa, A. lyrata ssp. lyrata, and A. lyrata ssp. petraea, respectively. Two pairs of tandem repeats were found in A. thaliana. No tandem repeat was found A. halleri. (0.08 MB PDF) [file pgen.1000048.s003.pdf]

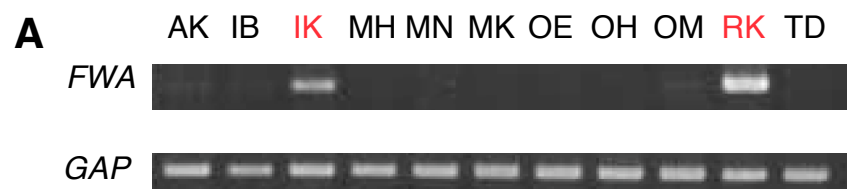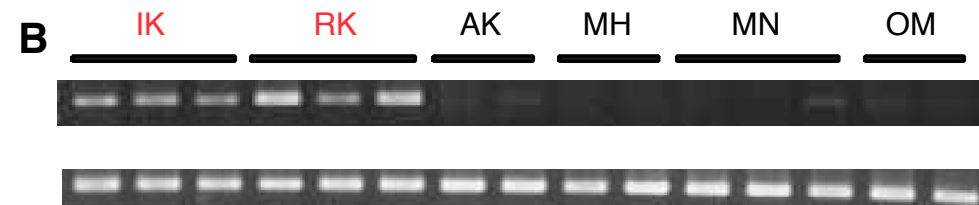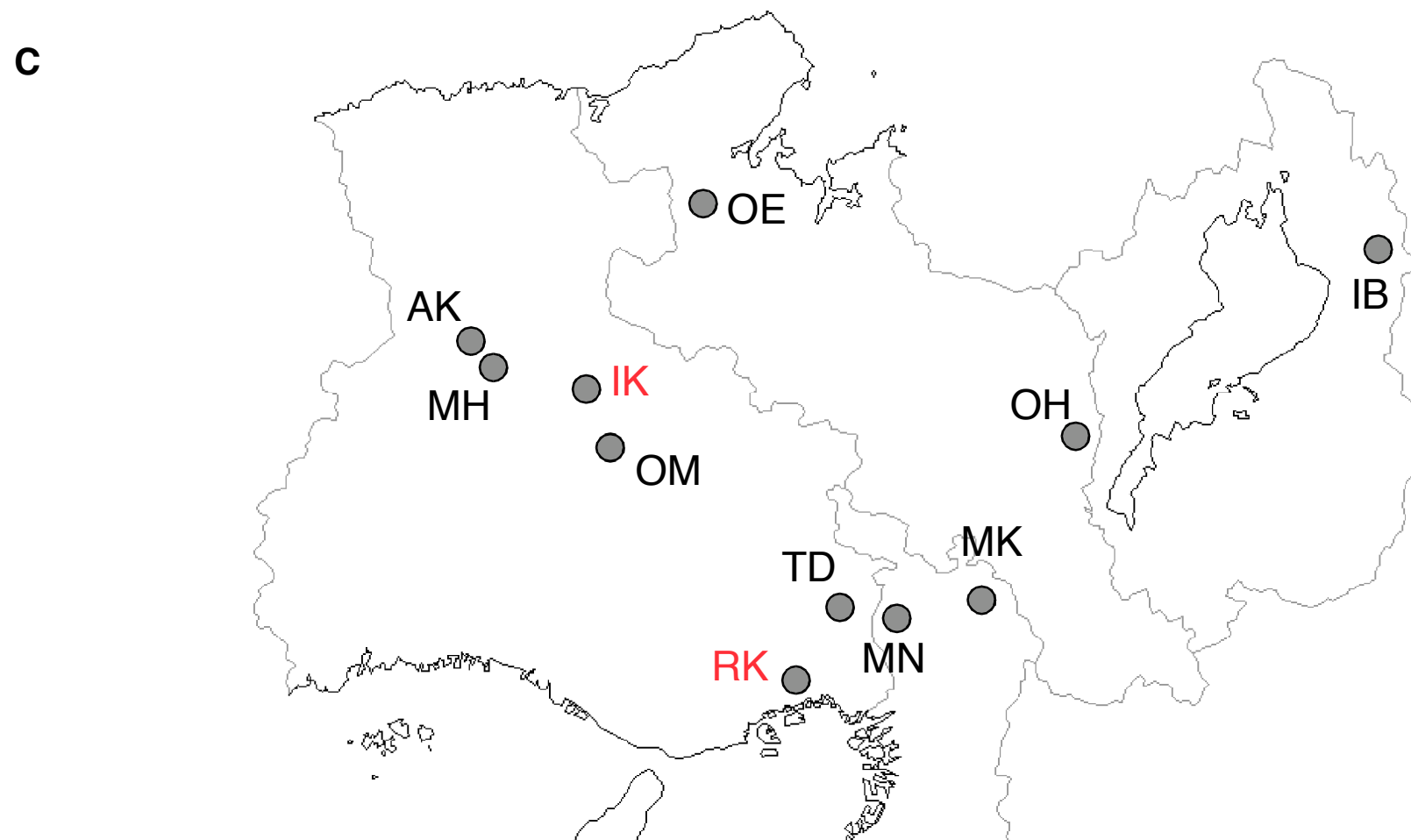

Supplement: Figure S3 — Variation in vegetative FWA expression in eleven plants of A. halleri ssp. gemmifera isolated around Kyoto area in Japan. (A) The vegetative FWA expression was detectable in two isolates, while it was undetectable in the other nine isolates. (B) Same expression pattern was detected in different individuals isolated from the same area. (C) Map of the locations these plants were isolated. Abbreviation of the isolates: AK (Akebe), MH (Mikohata), IK (Ikuno), OM (Omoidegawa, Taka), RK (Rokko), TD (Tada), MK (Myouken), MN (Mino), OH (Ohara), IB (Mt. Ibuki), OE (Mt. Ooe). The plants were isolated from the field and were grown in the laboratory. Three of them, Mino, Omoidegawa, and Tada, were further characterized after isolation of the seeds and growth in the laboratory (Figure 3B). (0.12 MB PDF) [file pgen.1000048.s004.pdf]

**A**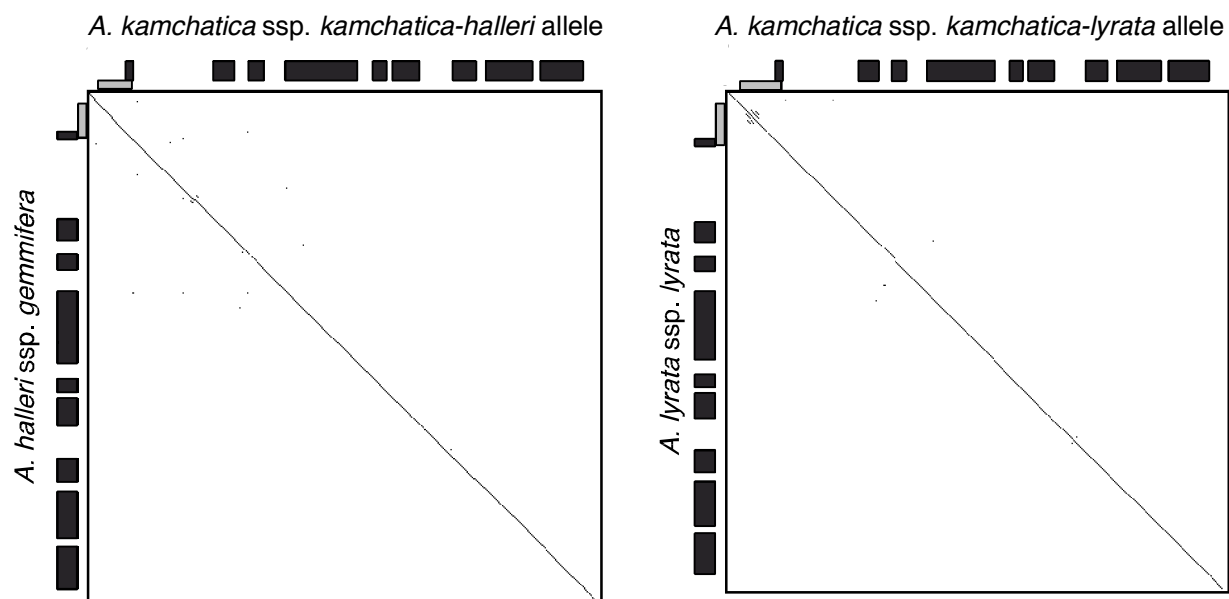**B**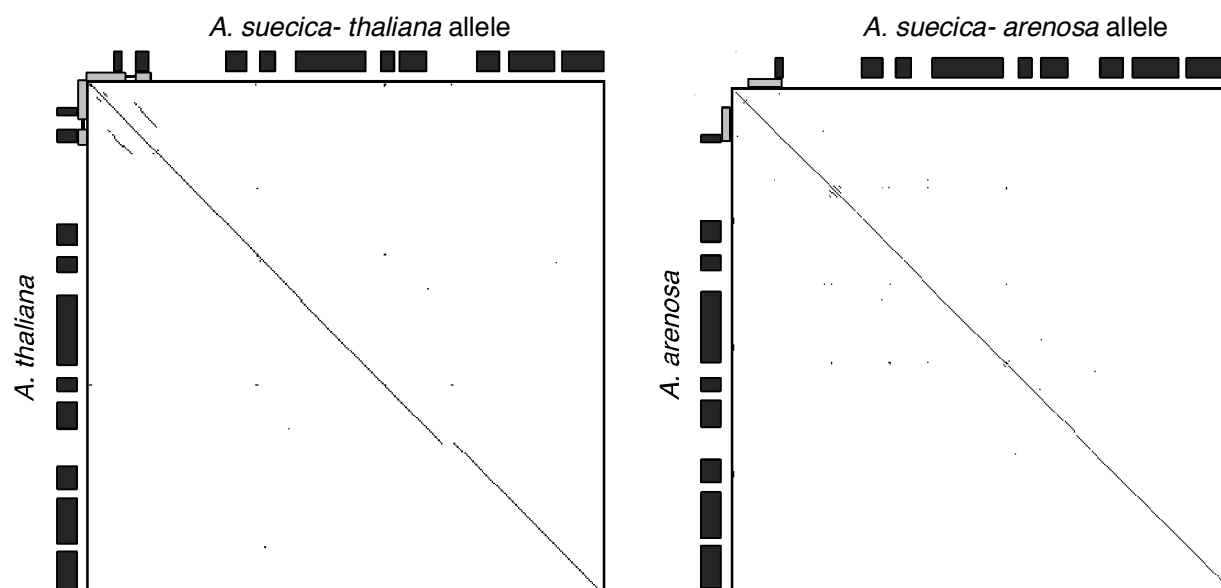**C**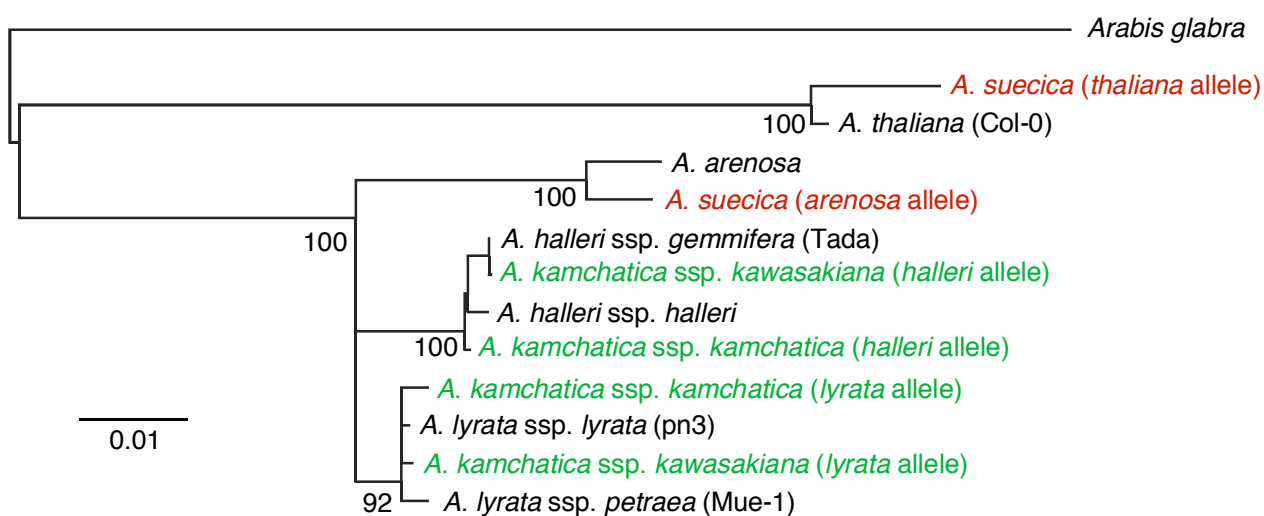

Supplement: Figure S4 — Structure of FWA genes in allotetraploids and parental species. (A) Two FWA copies in A. kamchatica. Each of them was similar to that in A. halleri (left panel) or A. lyrata (right panel). The similarity was found not only in exons (black boxes) but also promoter and introns. Gray boxes indicate the SINE-related regions. Copy number of tandem repeats in the lyrata-like copy was three as is the case in ssp. lyrata. (B) Two FWA copies in A. suecica. Each of them was similar to that in A. thaliana (left panel) or A. arenosa (right panel). (C) A phylogenetic tree of nucleotide sequences of full length cDNA of FWA in the Arabidopsis species constructed by the neighbor-joining method. Arabis glabra FWA was used as the outgroup. Bootstrap values with 1,000 repeats are indicated at the nodes of the neighbor-joining tree. (0.08 MB PDF) [file pgen.1000048.s005.pdf]

**A**

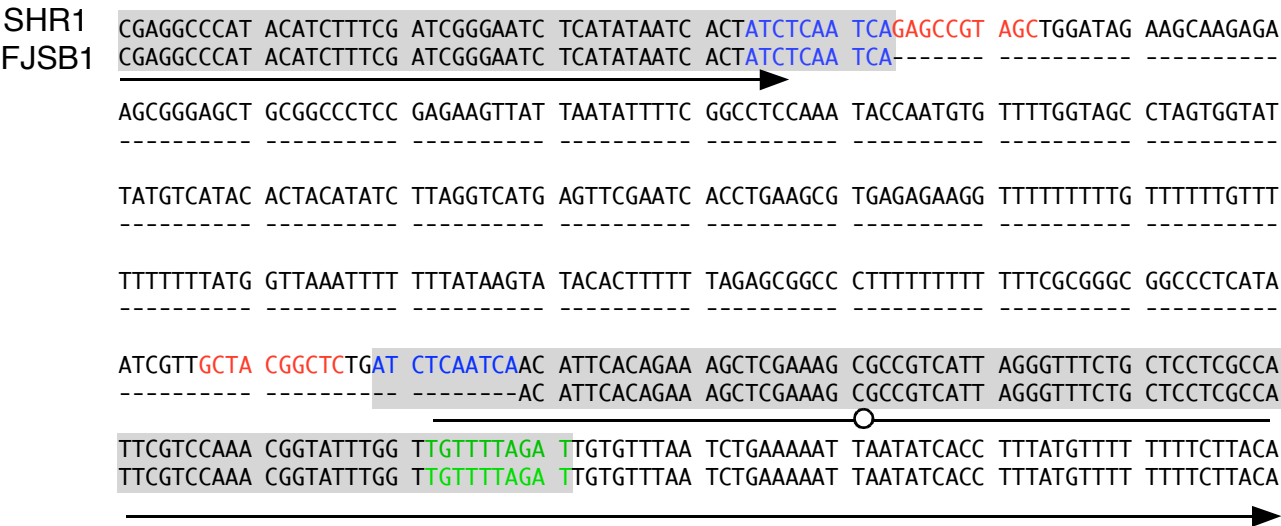

**B**

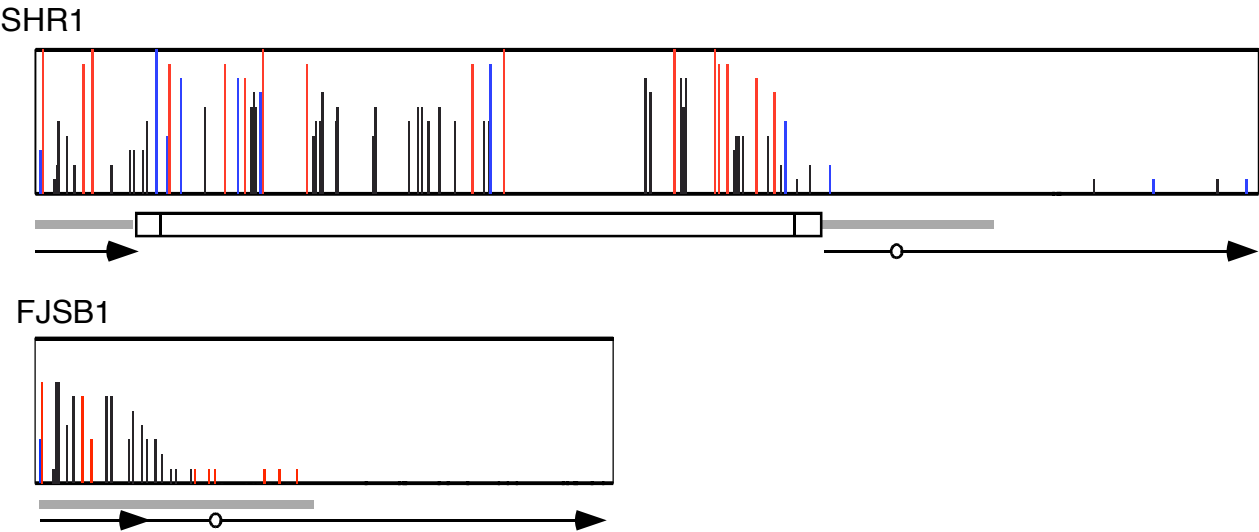

**C**

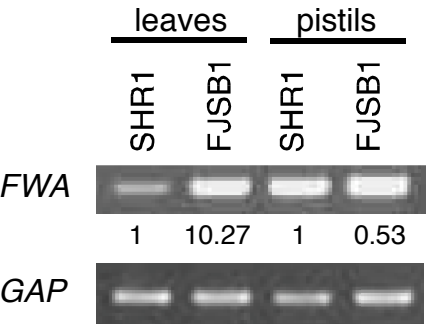

Supplement: Figure S5 — Differences in promoter structure and expression pattern of the halleri-type FWA gene in two strains of in A. kamchatica, SHR1 (ssp. kawasakiana), and FJSB1 (ssp. kamchatica). (A) Promoter alignment revealed insertion of a possible transposable element in the SHR1 allele. Blue and red letters indicate the predicted target site duplication and terminal inverted repeat, respectively. This insertion was found in majority of A. kamchatica, ssp. kawasakiana strains isolated in Japan (not shown). Gray boxes: the SINE-related sequences. Green letter shows the target site duplication of the SINE insertion. The circle shows the transcription start site. Black arrows show regions duplicated in A. thaliana. (B) Methylation status of the halleri-type FWA gene in the two strains. The predicted transposon (white box) was heavily methylated in SHR1. (C) Expression analysis of the FWA gene. Related FWA expression level measured by real-time PCR are shown below the gel. The transposon insertion and increase in the DNA methylation correlates with reduction in the FWA transcript level in SHR1. (0.04 MB PDF) [file pgen.1000048.s006.pdf]

Between *A. thaliana* and *A. halleri* ssp. *gemmifera*

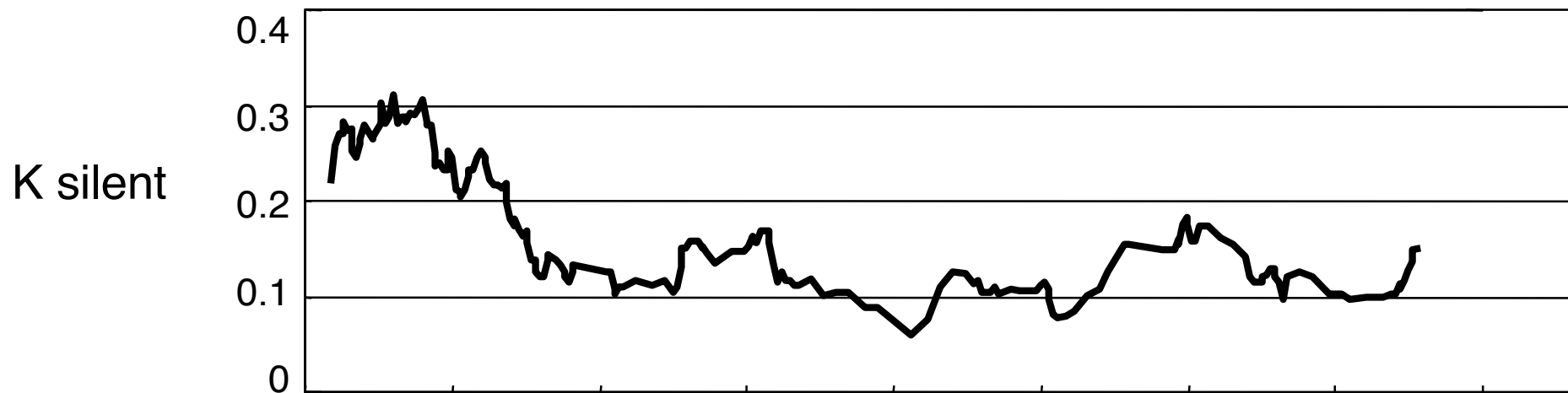

Within *A. thaliana*

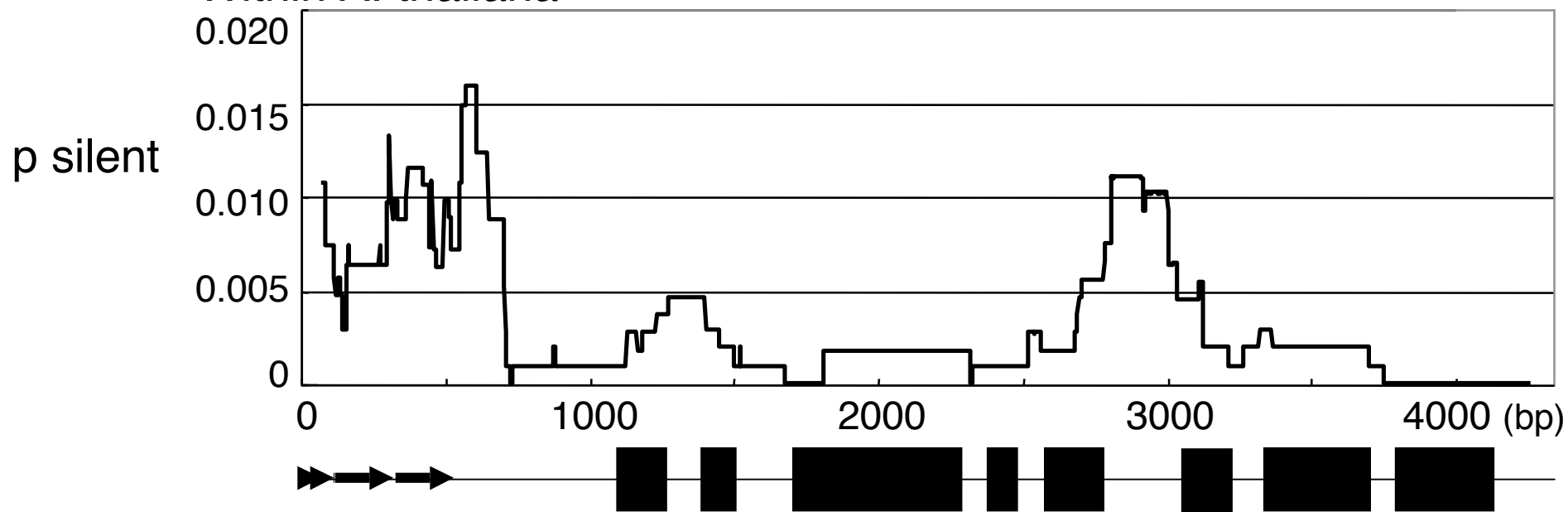

Supplement: Figure S6 — Sliding window analyses of silent divergence and diversity in the FWA gene. The analyses are based on the 12 strains of A. thaliana. K and p in 200 bp silent sites are plotted in 1 bp intervals. Under the sliding window plot, exons (from third to last) are shown by black boxes and repeated regions by arrows. (0.02 MB PDF) [file pgen.1000048.s007.pdf]
