# Supplementary material for: Evolution and Control of Imprinted FWA Genes in the Genus Arabidopsis
Source: PLoS Genet. 2008 Apr 4;4(4):e1000048. doi: 10.1371/journal.pgen.1000048 (PMC2270340; doi:10.1371/journal.pgen.1000048)
Supplement: Table S1 — Proportion of methylated cytosine around the FWA promoter. (0.04 MB PDF) [file pgen.1000048.s008.pdf]

Supplementary Table S1

Proportion of methylated cytosine around the *FWA* promoter.

	At [a]	*Aa* [b]	*Al-l* [c] (pn3)	*Al-l* [c] (MN47)	*Al-p* [d]	*Ah-g* [e]	*Ah-h* [f]	*Ah-t* [g]	*Ah-o* [h]
Upstream [i]									
CG	88.57	93.33	54.00	72.00	97.14	96.00	100.00	100.00	92.00
non-CG	33.85	10.59	24.24	54.85	48.46	50.59	38.24	16.47	34.71
Total	45.45	32.17	28.16	57.11	55.87	60.91	52.27	35.45	47.73
Downstream [j]									
CG	77.14	73.33	0.00	3.33	85.00	48.57	85.71	91.43	70.00
non-CG	36.73	1.30	0.00	2.50	10.50	13.04	8.26	5.65	7.50
Total	44.93	16.21	0.00	2.69	27.69	21.33	26.33	25.67	21.61

The values were calculated from the results shown in Figure 6. [a]*A. thaliana*. [b]*A.arenosa*. [c]*A.lyrata* ssp. *lyrata.* [d]*A.lyrata* ssp. *petraea*. [e]*A.halleri* ssp. *gemmifera*. [f]*A.halleri* ssp. *halleri.* [g]*A.halleri* ssp. *tatlica*. [h]*A.halleri* ssp. *ovirensis*. [i]Upstream of the transcription start site (TSS). [j]Downstream of the TSS.
