# Supplementary material for: Evolution and Control of Imprinted FWA Genes in the Genus Arabidopsis
Source: PLoS Genet. 2008 Apr 4;4(4):e1000048. doi: 10.1371/journal.pgen.1000048 (PMC2270340; doi:10.1371/journal.pgen.1000048)
Supplement: Table S2 — Patterns of nucleotide variation in the FWA gene within A. thaliana. (0.05 MB PDF) [file pgen.1000048.s009.pdf]

Supplementary Table S2

Patterns of nucleotide variation in the *FWA* gene within *A. thaliana*

							Direction of mutations in *A. thaliana* [c]			
	N[a]	Length	GC contents	K [b]	π	Tajima's D	AT to GC	GC to AT	AT to AT	GC to GC
Direct repeat region	96	474	0.354	0.2187	0.0079	-1.873 *	7 (D=-1.564 *)	27 (D=-1.708 *)	4	3
	12	521	0.354	0.2120	0.0082	-1.007	1	15	0	2
Other regions										
All sites	12	3609	0.380	0.1212	0.0022	-1.426	16	16	7	2
Silent sites	12	2029.90		0.1265	0.0030	-1.331	13	11	5	2
Noncoding region	12	1551	0.313	0.1344	0.0034	-1.244	11	10	3	2
Synonymous sites	12	478.90		0.1052	0.0017	-1.357	2	1	2	0
Replacement sites	12	1579.10		0.1155	0.0012	-1.499	3	5	2	0

Ratios of mutation direction between AT to GC and GC to AT significantly differ between repeat region and other region ($\chi 2 = 6.24$, $p = 0.001$).

[a]: Sample size used for the sequence analyses. [b]: Divergence between *A. thaliana* and *A. halleri* ssp. *gemmifera*. [c]: Directions of the mutations were determined parsimoniously using *A. halleri* ssp. *gemmifera* and the other copy of the repeat sequences. When these two sequences differ in variable sites, nucleotide of the other copy of the repeat was used as the ancestral state. Within two classes of them, Tajima's D was calculated as shown in parenthesis.

*: $p < 0.05$.
